# Pulmonary Renal Syndrome in ANCA-Negative Vasculitis

**DOI:** 10.7759/cureus.52491

**Published:** 2024-01-18

**Authors:** Ijeoma Orabueze, Hira Sheikh, Valerie Cluzet

**Affiliations:** 1 Internal Medicine, Vassar Brothers Medical Center, Poughkeepsie, USA; 2 Infectious Diseases, Vassar Brothers Medical Center, Poughkeepsie, USA

**Keywords:** anca-negative vasculitis, pulmonary renal syndrome, diffuse alveolar hemorrhage, plasmapheresis, anca vasculitis, hemodialysis

## Abstract

Below we highlight a rare case of anti-neutrophil cytoplasmic antibody (ANCA)-negative vasculitis, unique in its own right, as the diagnosis was hard to make and the respiratory decline rapid, with the patient going from a 23% fraction of inspired oxygen (FiO_2_) on admission to 100% FiO_2_ within four days for what was initially presumed to be community-acquired pneumonia. Precise data on the incidence or prevalence of ANCA-associated vasculitis are lacking. However, a 20-year population-based study in the United States found that, of 58 incident cases, 9% were ANCA-negative. We present the case of a 69-year-old Egyptian male with worsening shortness of breath who was found to have elevated inflammatory markers and an ANCA-negative panel and was later diagnosed with ANCA-negative vasculitis. By highlighting this case, we aim to increase awareness and point out the need to keep the disease high on the list of differential diagnoses in order to allow for timely intervention. Though there isn't a lot of data available on definitive treatment or the disease itself, there are studies that point to rituximab, cyclophosphamide, plasmapheresis, and hemodialysis as useful interventions for treatment.

## Introduction

Vasculitis is characterized by inflammation and necrosis involving a wide spectrum of vasculatures of various sizes and locations [1]. The absence of specific markers in seronegative anti-neutrophil cytoplasmic antibody (ANCA) vasculitis may lead to a delay in diagnosis or misdiagnosis; a definitive diagnosis requires histologic confirmation. A 20-year population-based study calculated the annual incidence of ANCA-negative vasculitis to be 0.3% [2]. Although not common, pulmonary renal syndrome (PRS) in the form of diffuse alveolar hemorrhage (DAH) can occur in patients with ANCA-negative vasculitis.

## Case presentation

A 69-year-old male with chronic back pain status post-laminectomy, on a morphine pump, with hypertension and dyslipidemia, presented to the emergency department with worsening shortness of breath and anorexia for five days. Upon arrival, he had a temperature of 99.5F, was tachypneic, and was hypoxic. The physical exam was notable for bibasilar rales. Initial laboratory values revealed mild leukocytosis (14*10 (9)/l), D-dimer of 2376 ng/mL, mild hyponatremia of 134 mmol/l (135-145 mmol/l), N-terminal pro-B-type natriuretic peptide (NT-proBNP) of 1,248 pg/mL (<=299 pg/ml), and troponin of 185 ng/L (<=22 ng/l).

**Figure 1:**
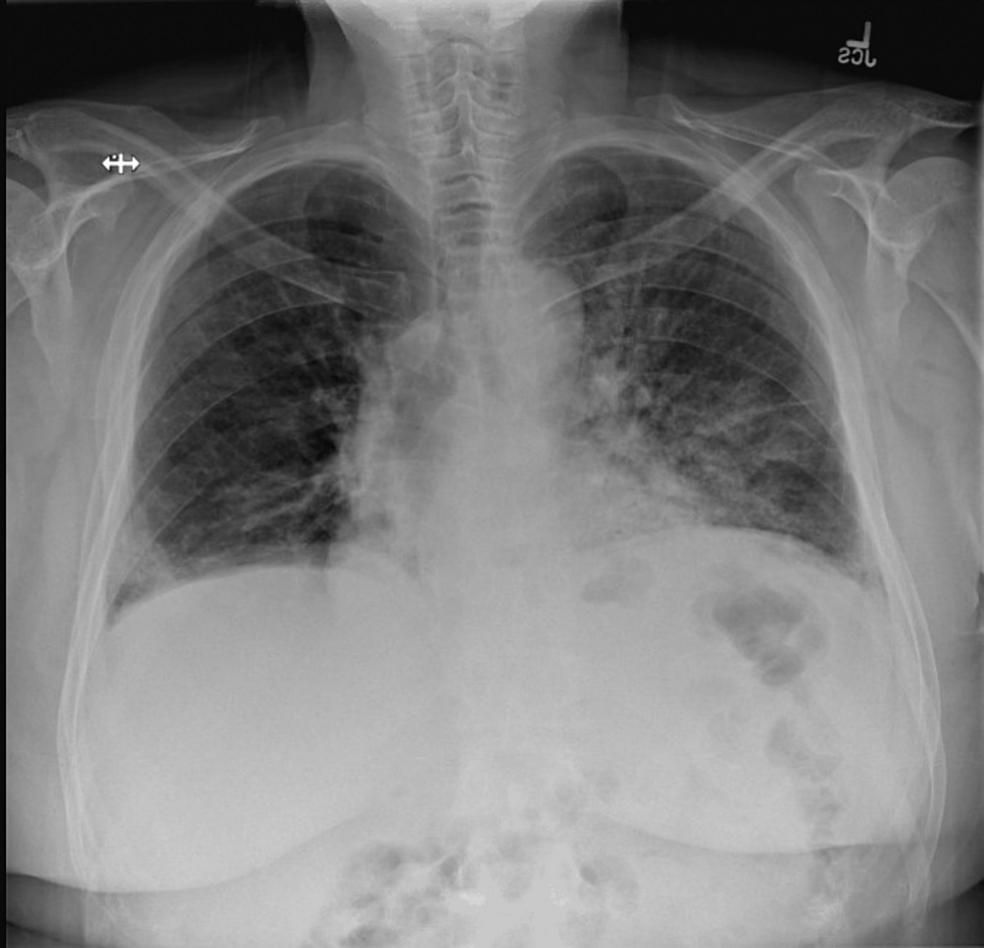
Chest X-ray on admission showed more perihilar interstitial prominence on the left than on the right.

**Figure 2:**
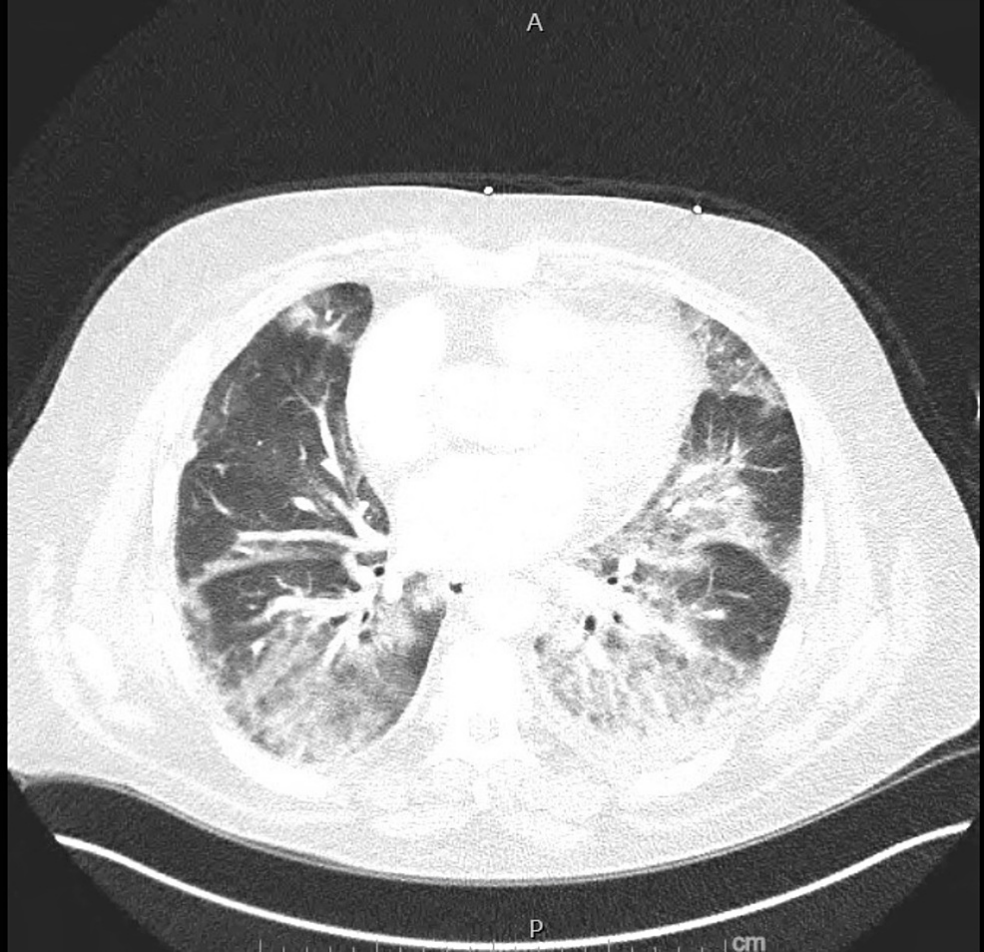
CT angiography of the chest This was performed due to elevated D-dimer levels to rule out a pulmonary embolus. There was no evidence of pulmonary embolism, but it showed diffuse bilateral lung infiltrates, similar to the findings on the chest X-ray.

The chest X-ray (Figure 1) and chest CT (Figure 2) noted more perihilar interstitial prominence on the left than on the right.

**Figure 3:**
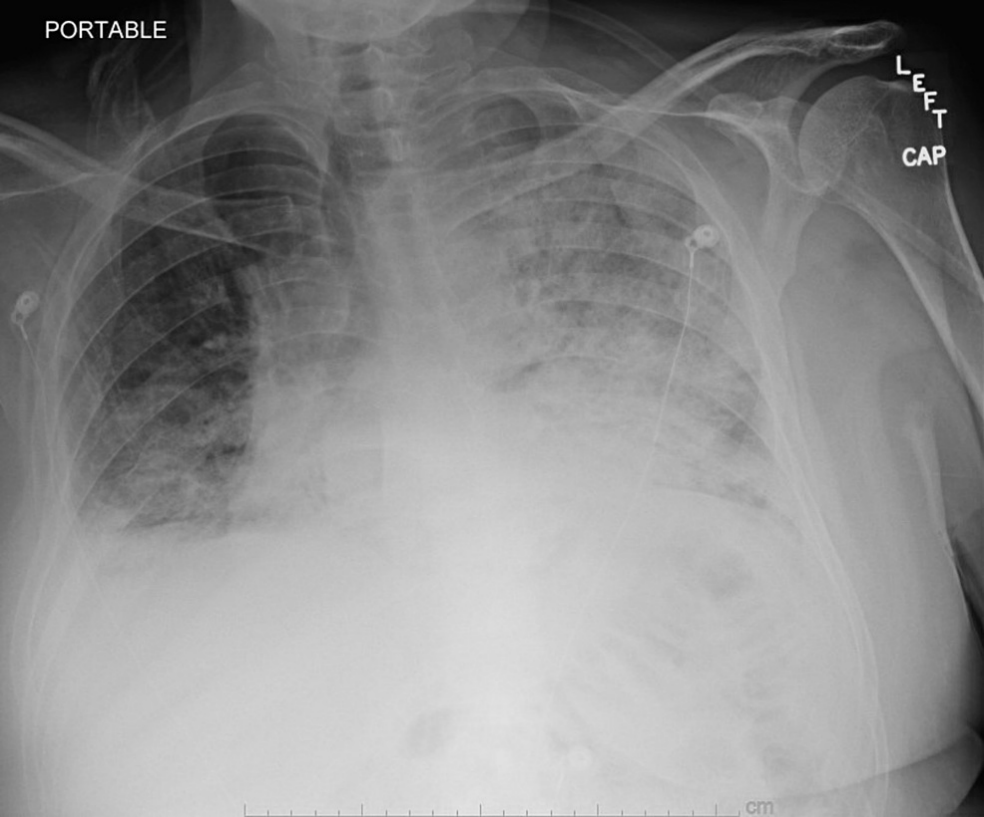
Chest X-ray performed four days after admission This was performed due to the patient's increased oxygen requirements. There is a significant worsening of the bilateral infiltrates, with more on the left compared to the right.

The viral panel was negative. He was initially treated for community-acquired pneumonia with ceftriaxone and azithromycin. Pulmonology was consulted four days later due to increasing oxygen requirements, and a repeat chest X-ray (Figure 3) showed worsening bilateral infiltrates.

He was started on intravenous dexamethasone 6 mg daily. Despite this, his respiratory status worsened. Due to persistently elevated inflammatory markers (c-reactive protein (CRP): 280 mg/L (0.0-8 mg/l) and ferritin: 763 ng/ml (24-370 ng/ml), autoimmune etiologies were considered. Urinalysis revealed 30 mg/dl of protein, no red blood cell casts, and 0-2 red blood cells. Cytoplasmic-ANCA, perinuclear-ANCA, rheumatoid factor, anti-cyclin citrullinated peptide (CCP), anti-Sjögren's syndrome-related antigen A (anti-SSA), anti-Sjögren's syndrome-related antigen B (anti-SSB), antinuclear antibody (ANA), anti-glomerular basement membrane (anti-GBM), and anti-ribonucleoprotein (RNP) were all negative. Infectious workups for tuberculosis, histoplasma, aspergillus, *Pneumocystis jiroveci* pneumonia (PJP), and urine legionella antigen were negative. The patient’s renal function began to deteriorate as his creatinine uptrended to 3 mg/dl from a baseline of 0.86 mg/dl, so PRS was considered, and he was started on 250 mg of methylprednisolone every six hours. His respiratory status worsened, requiring intubation, and he underwent bronchoscopy, showing sequential bloody urine lavage, consistent with DAH. A kidney biopsy was considered at this point but was deferred as the patient was hemodynamically unstable on two vasopressors.

Given this clinical picture, ANCA-negative vasculitis was suspected, and cyclophosphamide was administered. Due to a lack of improvement, he underwent five sessions of plasma exchange. He required hemodialysis, and his renal function rapidly improved after three sessions. Unfortunately, due to a severe illness, the patient expired during hospitalization.

## Discussion

Pulmonary renal syndrome is defined as the combination of DAH and glomerulonephritis [3]. Pulmonary vasculitis, extravascular inflammation, and fibrosis are frequent components of ANCA vasculitis [4]. However, PRS in the form of DAH is not limited to ANCA-associated vasculitis but can occur in patients with ANCA-negative vasculitis [5]. Pulmonary renal syndrome has been described only occasionally in Behçet's disease, in Henoch-Schönlein purpura (HSP), and mixed cryoglobulinemia [3]. Our patient had no arthralgia or arthritis, abdominal pain, or bilateral lower extremity purpura to suggest HSP. Likewise, there were no oral aphthous ulcers, urogenital ulcers, cutaneous lesions like palpable purpura, pustular eruptions, or uveitis to suggest Behcet's disease. Furthermore, Behcet's disease typically affects patients in the age range of 20-40 years, typically those of Turkish descent. Pulmonary renal syndrome presents a major challenge for management, particularly in the intensive care unit (ICU), as the initial diagnosis is ‘pneumonia' due to pulmonary infiltrates and fever and about a third of the ICU patients present with severe renal impairment or acute respiratory distress syndrome. It is therefore important to note that pneumonia may trigger PRS [3].

**Figure 4:**
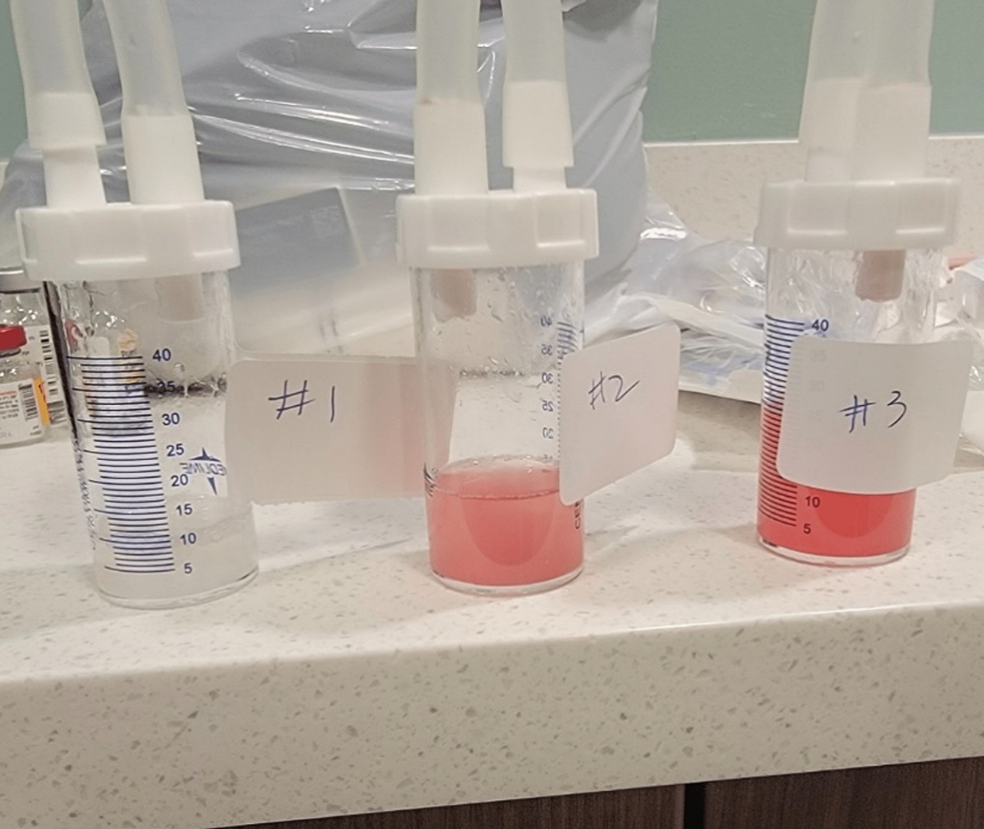
Bronchoscopy samples There is an increasing amount of blood in the three sequential lavage samples.

Pulmonary involvement in PRS is a small-vessel vasculitis whereby the arterioles, venules, and pulmonary capillaries are destroyed by an inflammatory process. This disrupts the continuity of the vessels, causing extravasation of blood and, hence, alveolar hemorrhage [3]. The renal involvement in PRS may manifest as focal proliferative glomerulonephritis, necrotizing granulomas, or small vessel vasculitis, which may be distinguished with the use of immunofluorescence [3]. The pathogenesis of ANCA-negative pauci-immune crescentic glomerulonephritis (CrGN) is not clear. It was found by Eisenberger et al. [6] that in patients with ANCA-negative pauci-immune CrGN, neutrophil infiltration could be found in glomerular lesions; therefore, neutrophils might play a pathogenic role even in the absence of ANCA [6]. The involvement of other mechanisms, such as lymphocytes or unidentified autoantibodies, in the pathogenesis of ANCA-negative pauci-immune CrGN needs further investigation. A renal biopsy would have been diagnostic in our patient, but this was deferred due to the patient requiring multiple pressors. Diagnosis of diffuse alveolar hemorrhage includes bronchoscopic examination for three purposes, which are to document alveolar hemorrhage by bronchoalveolar lavage, to exclude airway sources of bleeding by visual inspection, and to exclude an associated infection [7]. Diffuse alveolar hemorrhage is characterized by an increasing amount of blood on three sequential lavage samples. The specimens obtained from bronchoalveolar lavage (BAL) are demonstrated in the image (Figure 4).

In comparison to ANCA-associated vasculitis, ANCA-negative patients have a shorter interval from onset to diagnosis, a greater degree of proteinuria, a higher prevalence of nephrotic syndrome, and, on histology, more severe glomerular lesions [6]. Although a biopsy could not be performed due to the patient’s clinical status, this diagnosis was ultimately suspected due to the consistent clinical picture. It is important to initiate appropriate treatment as soon as ANCA-negative vasculitis is suspected, even in the absence of histopathological confirmation, in order to reduce morbidity and mortality [3]. Some articles have recommended the use of corticosteroids, cyclophosphamide, and plasma exchange for severe disease defined as serum creatinine above 5.7 mg/dl to improve renal function in ANCA-associated PRS [3]. Although ANCA specificity predicts differences in manifestations and response to therapy, little is known about the ideal treatment for ANCA-negative vasculitis, given its rarity.

## Conclusions

Making a diagnosis of pulmonary vasculitis is challenging. A correct and timely diagnosis is pivotal to initiating therapy early as the disease rapidly deteriorates. Our case demonstrates that appropriate diagnosis in the setting of primary pulmonary involvement remains a challenge and that hemodialysis may help with improving renal function. Initial laboratory evaluation should include inflammatory markers, renal and liver function tests, and the determination of ANCA.
